# Age at cancer diagnosis by breed, weight, sex, and cancer type in a cohort of more than 3,000 dogs: Determining the optimal age to initiate cancer screening in canine patients

**DOI:** 10.1371/journal.pone.0280795

**Published:** 2023-02-01

**Authors:** Jill M. Rafalko, Kristina M. Kruglyak, Angela L. McCleary-Wheeler, Vidit Goyal, Ashley Phelps-Dunn, Lilian K. Wong, Chelsea D. Warren, Gina Brandstetter, Michelle C. Rosentel, Lauren DiMarzio, Lisa M. McLennan, Allison L. O’Kell, Todd A. Cohen, Daniel S. Grosu, Jason Chibuk, Dana W. Y. Tsui, Ilya Chorny, Andi Flory

**Affiliations:** 1 Medical & Clinical Affairs, PetDx, La Jolla, California, United States of America; 2 Information Technology & Bioinformatics, PetDx, La Jolla, California, United States of America; 3 Research & Development, PetDx, La Jolla, California, United States of America; 4 Clinical Studies, PetDx, La Jolla, California, United States of America; 5 Chief Executive Officer, PetDx, La Jolla, California, United States of America; Bauer Research Foundation, UNITED STATES

## Abstract

The goal of cancer screening is to detect disease at an early stage when treatment may be more effective. Cancer screening in dogs has relied upon annual physical examinations and routine laboratory tests, which are largely inadequate for detecting preclinical disease. With the introduction of non-invasive liquid biopsy cancer detection methods, the discussion is shifting from *how* to screen dogs for cancer to *when* to screen dogs for cancer. To address this question, we analyzed data from 3,452 cancer-diagnosed dogs to determine the age at which dogs of certain breeds and weights are typically diagnosed with cancer. In our study population, the median age at cancer diagnosis was 8.8 years, with males diagnosed at younger ages than females, and neutered dogs diagnosed at significantly later ages than intact dogs. Overall, weight was inversely correlated with age at cancer diagnosis, and purebred dogs were diagnosed at significantly younger ages than mixed-breed dogs. For breeds represented by ≥10 dogs, a breed-based median age at diagnosis was calculated. A weight-based linear regression model was developed to predict the median age at diagnosis for breeds represented by ≤10 dogs and for mixed-breed dogs. Our findings, combined with findings from previous studies which established a long duration of the preclinical phase of cancer development in dogs, suggest that it might be reasonable to consider annual cancer screening starting 2 years prior to the median age at cancer diagnosis for dogs of similar breed or weight. This logic would support a general recommendation to start cancer screening for all dogs at the age of 7, and as early as age 4 for breeds with a lower median age at cancer diagnosis, in order to increase the likelihood of early detection and treatment.

## Introduction

Cancer is by far the leading cause of death in adult dogs [1]. The lifetime risk of cancer as well as cancer mortality in dogs are known to vary significantly by breed [2–4]. For example, ~50% of Irish Water Spaniels and Flat-Coated Retrievers die of cancer, whereas cancer-related mortality is significantly lower in breeds such as Shih Tzus and Dachshunds. However, even in the least-affected breeds, the rate of mortality from cancer is still 15–20% [3]. For comparison, common pathophysiologic processes, such as traumatic, infectious, metabolic, inflammatory, and degenerative, each account for ≤10% of deaths in adult dogs, across all breeds [1]. Cancer is thus a leading cause of death even among breeds that are relatively less affected by the disease, suggesting that all dogs–regardless of breed–would derive preventive benefit from regular cancer screening; yet options for canine cancer screening have historically been limited in comparison to the robust, guidelines-driven screening programs in human medicine [5]. We use the term screening here in the strict sense [6, 7], referring to measures taken to detect cancer preclinically in canine patients that are at higher risk for the disease because of their age or breed, but do not currently have clinical signs indicative of cancer.

Various veterinary professional organizations recognize the value of early cancer detection for optimizing outcomes [8–11], and veterinary academic institutions have issued prevention and screening recommendations for cancer in dogs [12, 13]. However, formal guidelines for earlier detection of cancer through regular screening programs do not exist in veterinary medicine as they do in human medicine [6, 14].

Liquid biopsy using next-generation sequencing of cell-free DNA has been introduced as a novel, non-invasive option for cancer screening in dogs [14, 15]. With the availability of a blood-based cancer test, the question of *how* to screen dogs for cancer may soon shift to *when* to start screening dogs for cancer. A “one age fits all” approach to the initiation of screening is unlikely to be appropriate for dogs, given the strong role of both genetic and environmental factors in the development of cancer and the great diversity of canine breeds and sizes.

Previous studies have focused on age at cancer diagnosis, or age at death from cancer, for individual breeds [16–18] or for specific cancer types [19–21], making the findings difficult to generalize to other breeds or to mixed-breed dogs. Some of the larger, population-based studies that incorporated a more diverse selection of breeds were conducted in Europe [2, 3, 22, 23] where common breeds may not be representative of breeds that are common in the United States; additionally, cancer incidence and cancer types observed in these studies may be different from those seen in a US population, given environmental differences and spay/neuter rates in Europe versus the United States.

We examined a large and heterogeneous population of cancer-diagnosed dogs, the vast majority of which were from the United States, representing >120 breeds and a wide variety of cancer types. The purpose of our study was to establish median ages at which dogs of various breeds and weights are diagnosed with cancer. Our findings, combined with previously published data regarding the duration of the preclinical phase of cancer, may assist in determining the age at which cancer screening should be initiated for individual dogs.

## Materials and methods

We evaluated data from 3,452 cancer-diagnosed dogs (herein “dogs”) sourced from 3 cohorts. Cohort 1 comprised 663 dogs prospectively enrolled in the CANcer Detection in Dogs (CANDiD) study [14]. All dogs were enrolled between 2019 and 2021 under protocols that received Institutional Animal Care and Use Committee (IACUC) or site-specific ethics approval, according to each site’s requirements. All dogs were client-owned, and written informed consent was obtained from all owners.

The dogs in cohort 1 were all-comers with a current definitive diagnosis of cancer (malignant neoplasia) of any type based on cytology and/or histology. All cancer-diagnosed dogs had complete staging, performed by the managing veterinarian according to standard-of-care staging guidelines at the enrolling site for that cancer type. A final review of all case forms and source documents was performed by the central study team (including board-certified veterinary medical oncologists) to ensure continuity of case details and to confirm the dog’s diagnosis by review of pathology records [14].

For dogs in cohort 1, the dog’s age (known or estimated; in years and months) at enrollment was provided by the enrolling veterinarian, along with the date of the patient’s cancer diagnosis. In the case of recurrence of a previous cancer, or a prior history of cancer (before the current diagnosis at enrollment in CANDiD), the date of diagnosis was the date of the first documented cancer diagnosis. Using the dog’s age, date of enrollment, and date of diagnosis, an age at diagnosis was calculated for all dogs.

Cohort 2 comprised 1,888 dogs from the National Cancer Institute (NCI), Division of Cancer Treatment and Diagnosis, Biological Testing Tumor Repository, deposited by the Canine Comparative Oncology Genetics Consortium [24]. The biospecimen repository was established in 2006. Samples were prospectively collected from academic institutions within the United States (Colorado State University, The Ohio State University, University of Wisconsin-Madison, Michigan State University, Tufts University, University of California-Davis, University of Missouri, University of Tennessee), and the standard operating procedures for the collection of samples were approved by each site’s IACUC.

The data from cohort 2 accompanied clinical samples from dogs with commonly diagnosed cancers, with a focus on enrolling dogs with 7 histologic diagnoses: osteosarcoma, lymphoma, malignant melanoma, hemangiosarcoma, soft tissue sarcoma, mast cell tumor, pulmonary tumor; however, dogs with other cancer types were not excluded from enrollment. Blood samples (collected from dogs prior to surgery) and tumor tissue were collected and submitted to the biorepository. As part of this process, clinical data regarding each dog, including patient demographics, pathology reports, treatment information, and longitudinal clinical follow-up were submitted to the NCI. In 2012, prior to the release of specimens to the research community, “quality control and quality assurance parameters were assessed on a panel of biospecimens distributed randomly across the 8 submitting institutions and the 7 tumor histologies represented in the repository” [24]; this included a comprehensive histologic review by a panel of board-certified veterinary pathologists from the NCI.

For cohort 2, the dog’s exact date of diagnosis or age at diagnosis were not collected as part of this dataset; however, an age at sample collection (in years) was documented for each dog. For the purpose of our study, the age at sample collection was used as a reasonable approximation for age at diagnosis, given that sample collection from the majority of dogs was expected to have occurred within weeks or, at most, months from the time the cancer diagnosis was first made. For cohorts 1 and 2, breed and weight information were provided for each dog by the veterinarian or staff at the enrolling site.

Cohort 3 was a subset of 901 dogs from a recent publication [25]. The dogs were patients of the Veterinary Medical Teaching Hospital (VMTH) at the University of California–Davis. Information about each dog was obtained via retrospective chart review and provided in the Supplementary Materials section of the publication [25]. According to the 2020 publication, “the study period represented 15 years of data for most breeds”. From the subset of dogs with cancer diagnoses in this study, we used the following data: breed, sex, neuter status, date of birth, date at cancer diagnosis (which allowed for calculation of an age at diagnosis), and cancer type (specifically, data was available for 4 common cancer types: lymphoma, mast cell tumor, osteosarcoma, hemangiosarcoma). Weight data were not available for any dogs in this cohort.

All dogs in cohort 3 received a cancer diagnosis at the VMTH, or were diagnosed by a referring veterinarian and the diagnosis was later confirmed at the VMTH. The diagnosis for each patient was confirmed by one or more of the following tests: chemistry panel, appropriate blood cell analyses, imaging, histopathology, and/or cytology, as described elsewhere [25]. If a diagnosis was listed in the record as suspected based on clinical signs, but not confirmed, the case was excluded.

In summary, the study population (cohorts 1, 2, and 3 combined) comprised dogs with a diagnosis of cancer, regardless of type, grade, or stage. Dogs with low-grade malignant tumors were not excluded from our study, as these tumors are commonly treated in oncology practice and are differentiated histologically from benign disease [26–28]. For our purposes, dogs were classified solely by their cancer type. No analysis was conducted based on grade or stage of disease, given that this information was not available for many dogs, and this type of analysis would be beyond the scope of our study.

Cases classified as malignant melanoma from cohort 1 included oral, ungual, anal sac, and cutaneous tumors. Melanomas included from cohort 2 included oral and cutaneous tumors, and as noted above, all cases had confirmed diagnoses of malignant tumors, per the study protocol [24]. Additionally, we grouped lymphomas and lymphoid leukemias for our study, because these diagnoses were not delineated in the underlying datasets, and “differentiating between true leukemia and stage V lymphoma can be difficult and arbitrary” [29]; furthermore, there is no staging system for acute lymphoid leukemia; hence, dogs were staged according to the lymphoma staging system as stage V. Histiocytic sarcoma was used as a specific diagnosis reserved for tumors under the broader category of histiocytic disease [30].

The overall study population (cohorts 1, 2, and 3 combined) was examined to determine the mean and median age at cancer diagnosis. Distributions were summarized by quartile and inter-quartile range (IQR). Categorical data were summarized as fractions. Age at cancer diagnosis was also analyzed within subsets by breed, weight, sex, and cancer type, with results summarized as mean and median age at diagnosis per category. For continuous variables, *p*-values were calculated using a Wilcoxon Rank-Sum test, and results with *p*<0.05 were considered statistically significant. When evaluating interaction between demographic variables on age at diagnosis, the *p*-value of the interaction term from the corresponding linear model was used, and results with *p*<0.05 were considered statistically significant (i.e., that an interaction between the demographic variables was observed). To evaluate the relationship between age at diagnosis and weight, a linear regression model was built to model median age at diagnosis as a function of weight brackets. The goodness of fit was evaluated based on the model coefficient of determination, *R*^2^; the significance of weight in predicting age at diagnosis was evaluated based on the *p*-value for the corresponding model coefficient. All analyses were performed using RStudio v2022.07.0.

## Results

### Demographics

The combined study population of 3,452 dogs comprised 2,537 dogs reported to be purebred, 858 reported to be mixed-breed, and 57 whose breed was described as other. Given that there was no significant difference between the ages at cancer diagnosis for dogs described as mixed-breed and other (*p* = 0.6560), these groups were combined for the purpose of data analysis, resulting in 915 dogs classified as mixed-breed or other.

The study population consisted of 1,900 males (55%) and 1,552 females (45%); 76% of males were castrated and 90% of females were spayed. Weight data were available for all 2,551 dogs from cohorts 1 and 2, and those dogs weighed 2.5–98.0 kg, with a mean of 30.3 kg and a median of 30.6 kg (Table 1). A full analysis of the demographics/characteristics of patients contributed by each of the three cohorts that provided data for the current study can be found in S1 Table.

**Table 1:** Demographics/Characteristics of the study population of cancer-diagnosed dogs.

Characteristics	Disposition of study population
Breed (n = 3,452)	Purebred: 2,537
	Mixed-breed or other: 915
Sex (n = 3,452)	Male: 1,900
	• Male (castrated): 1,452
	• Male (intact): 446
	• Male (status not provided): 2
	Female: 1,552
	• Female (spayed): 1,390
	• Female (intact): 161
	• Female (status not provided): 1
Weight* (n = 2,551)	Range: 2.5–98.0 kg
	Mean: 30.3 kg
	Median: 30.6 kg

* Weight data were not available for the 901 dogs in cohort 3.

Dogs were assigned a cancer type, based primarily on anatomic location, as described previously [14]. This classification system was adapted from elsewhere [29, 31]. The most common cancer type in the study population was lymphoma, followed by osteosarcoma, mast cell tumor, hemangiosarcoma, and soft tissue sarcoma (Table 2). There were 25 cases (from cohort 2) in which a diagnosis of malignancy was confirmed, but a cancer type could not be assigned given limited clinical information provided in the underlying dataset; the cancer type for these dogs was recorded as “Unknown” (Table 2). An analysis of the percent contributions of the three cohorts that provided data for the current study can be found in S2 Table.

**Table 2:** Cancer types represented in the study population of 3,452 dogs.

Cancer type and/or location	Number of dogs
Lymphoma/lymphoid leukemia	979
Bone, osteosarcoma	664
Mast cell tumor	565
Hemangiosarcoma	292
Soft tissue sarcoma	240
Malignant melanoma	128
Lung	113
Oral cavity	67
Skin	44
Histiocytic sarcoma	40
Peripheral nerve sheath	33
Anal sac adenocarcinoma	29
Multiple concurrent primary cancers	27
Unknown*	25
Chondrosarcoma	22
Liver	22
Urinary bladder/urethra	18
Nasal cavity and paranasal sinuses	16
Mammary gland carcinoma	15
Thyroid	15
Bone, multilobular osteochondrosarcoma	12
Bone, fibrosarcoma	9
Adrenal gland	8
Bone, sarcoma (other)	8
Brain	8
Spleen	8
Kidney	6
Small intestine	6
Prostate	5
Transmissible venereal tumor	5
Heart base	3
Pancreas	3
Bile duct	2
Mediastinum	2
Multiple myeloma	2
Salivary gland	2
Spinal cord	2
Ear canal	1
Esophagus	1
Large intestine	1
Nasal planum	1
Thymoma	1
Uterus	1
Vagina	1

Classification of cancer types was based primarily on anatomic location; adapted from Withrow and MacEwen’s Small Animal Clinical Oncology (Sixth Edition) and from the American Joint Committee on Cancer (AJCC) Manual (Eighth Edition).

* Diagnosis of malignancy was confirmed, but cancer type was not assigned given limited clinical information in the underlying dataset. All patients originated from cohort 2.

### Distribution of age groups at cancer diagnosis

**Fig 1:**
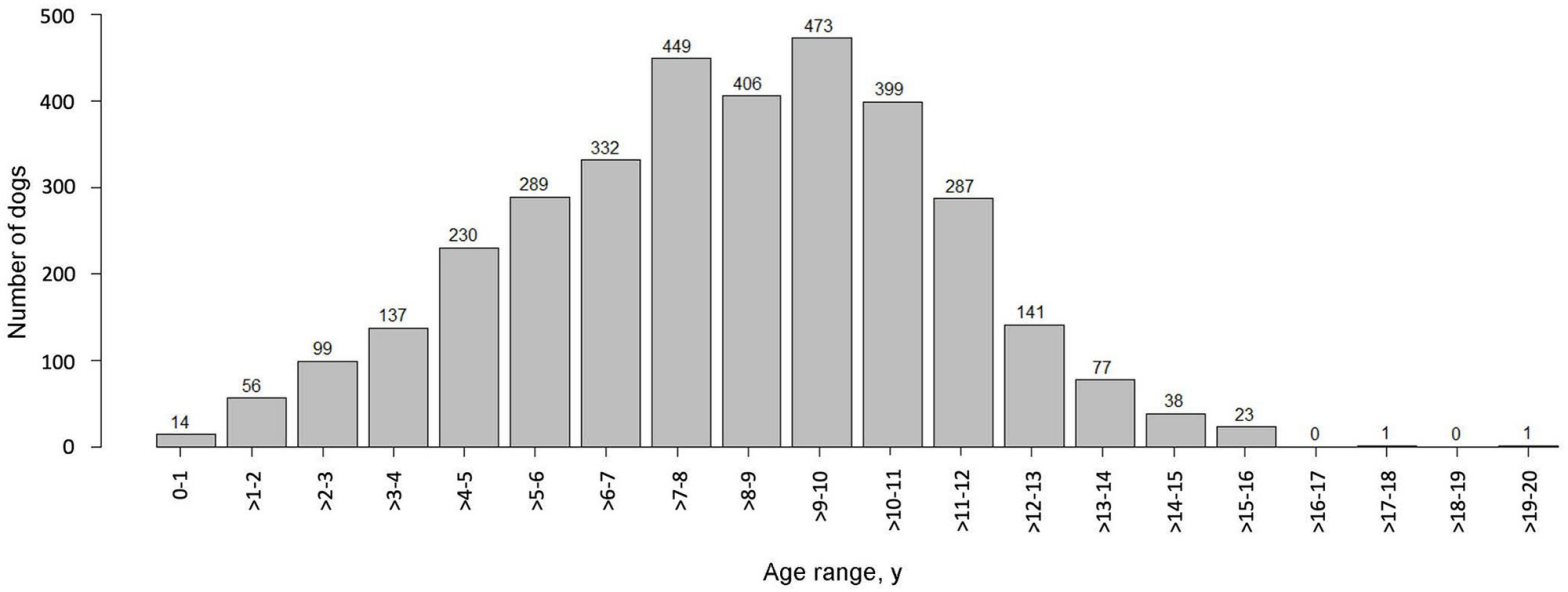
Distribution of 3,452 client-owned dogs by age at cancer diagnosis.

For the entire cohort of 3,452 dogs, the age at cancer diagnosis ranged from <1–20 years, with a mean of 8.5 years and a median of 8.8 years (Fig 1; S3 Table).

### Age at cancer diagnosis by breed

The age at cancer diagnosis for the 2,537 purebred dogs in our study ranged from <1–20 years of age, with a mean of 8.2 years and a median of 8.0 years. These dogs represented 122 distinct breeds (S4 Table). The breeds most highly represented were Golden Retrievers (*n* = 422) and Labrador Retrievers (*n* = 397), followed by Boxers (*n* = 178), Rottweilers (*n* = 168), and German Shepherds (*n* = 102).

**Fig 2:**
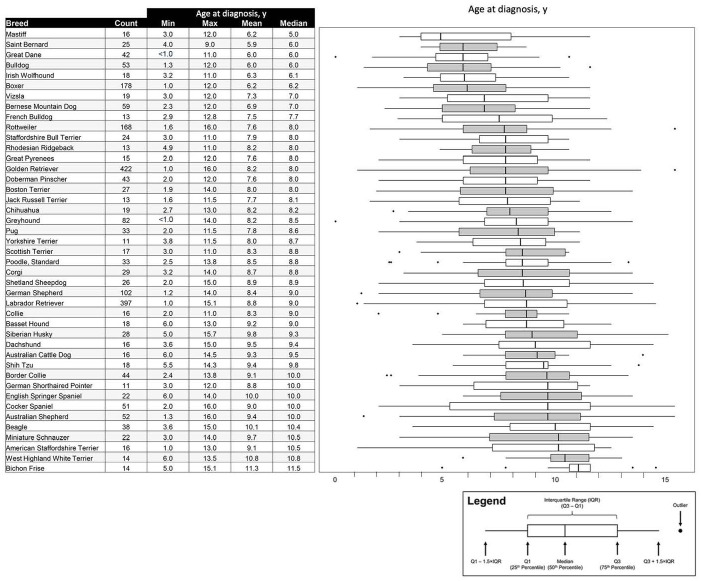
Age distribution at cancer diagnosis by breed for breeds represented by ≥10 dogs.

For the 43 breeds represented by ≥10 dogs, mean and median ages at diagnosis for the breed were calculated. The breeds with the youngest median age at cancer diagnosis were Mastiffs (median: 5 years), Saint Bernards, Great Danes, Bulldogs (median: 6 years), followed by Irish Wolfhounds (median 6.1 years), Boxers (median: 6.2 years), and Vizslas and Bernese Mountain Dogs (median: 7.0 years). The breed with the oldest median age at cancer diagnosis was the Bichon Frise (median: 11.5 years; Fig 2).

**Fig 3:**
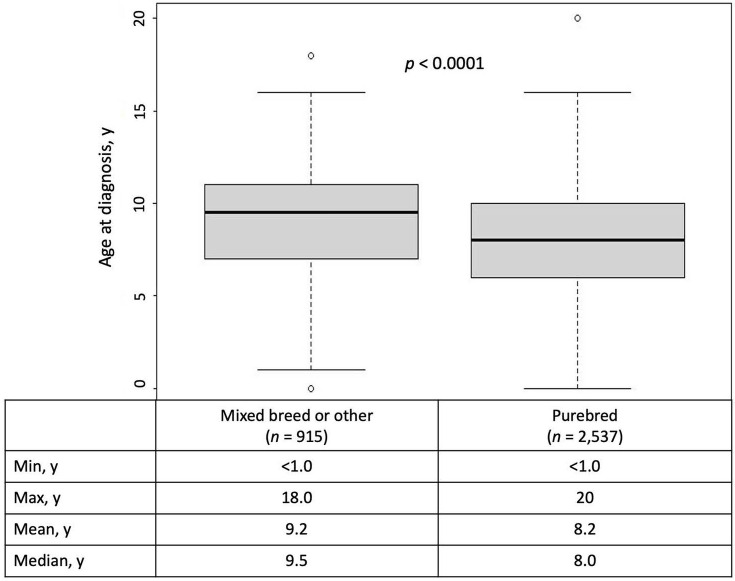
Age distribution at cancer diagnosis for mixed-breed or other vs. purebred dogs.

For mixed-breed or other dogs (*n* = 915), age at cancer diagnosis ranged from <1–18 years of age, with a mean of 9.2 years and a median of 9.5 years. The mean age at cancer diagnosis for these 915 mixed-breed or other dogs was significantly later than the mean age at diagnosis for the 2,537 purebred dogs in this study (9.2 vs. 8.2 years; *p* <0001; Fig 3). No interaction was observed between weight and pure- vs. mixed-breed status (*p* = 0.4907).

### Age at cancer diagnosis by weight

**Fig 4:**
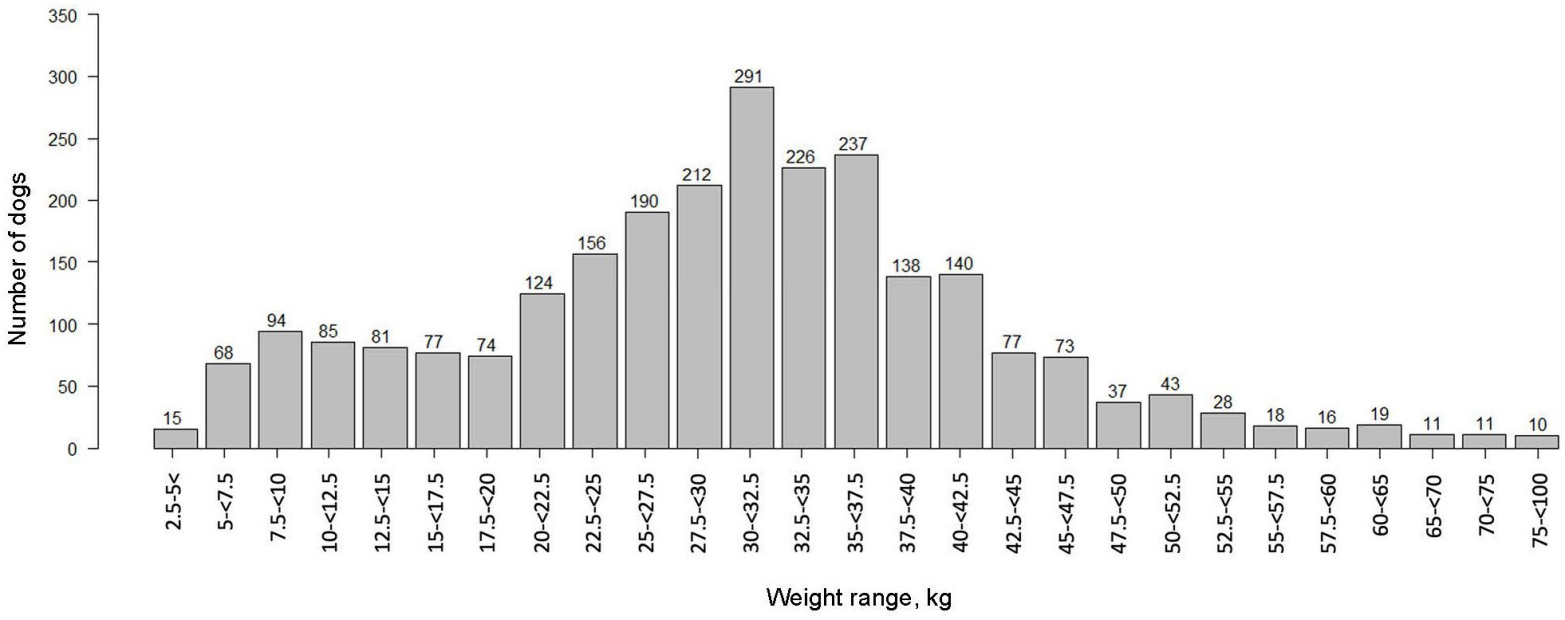
Weight distribution of the study population for 2,551 dogs that had a documented weight.

**Fig 5:**
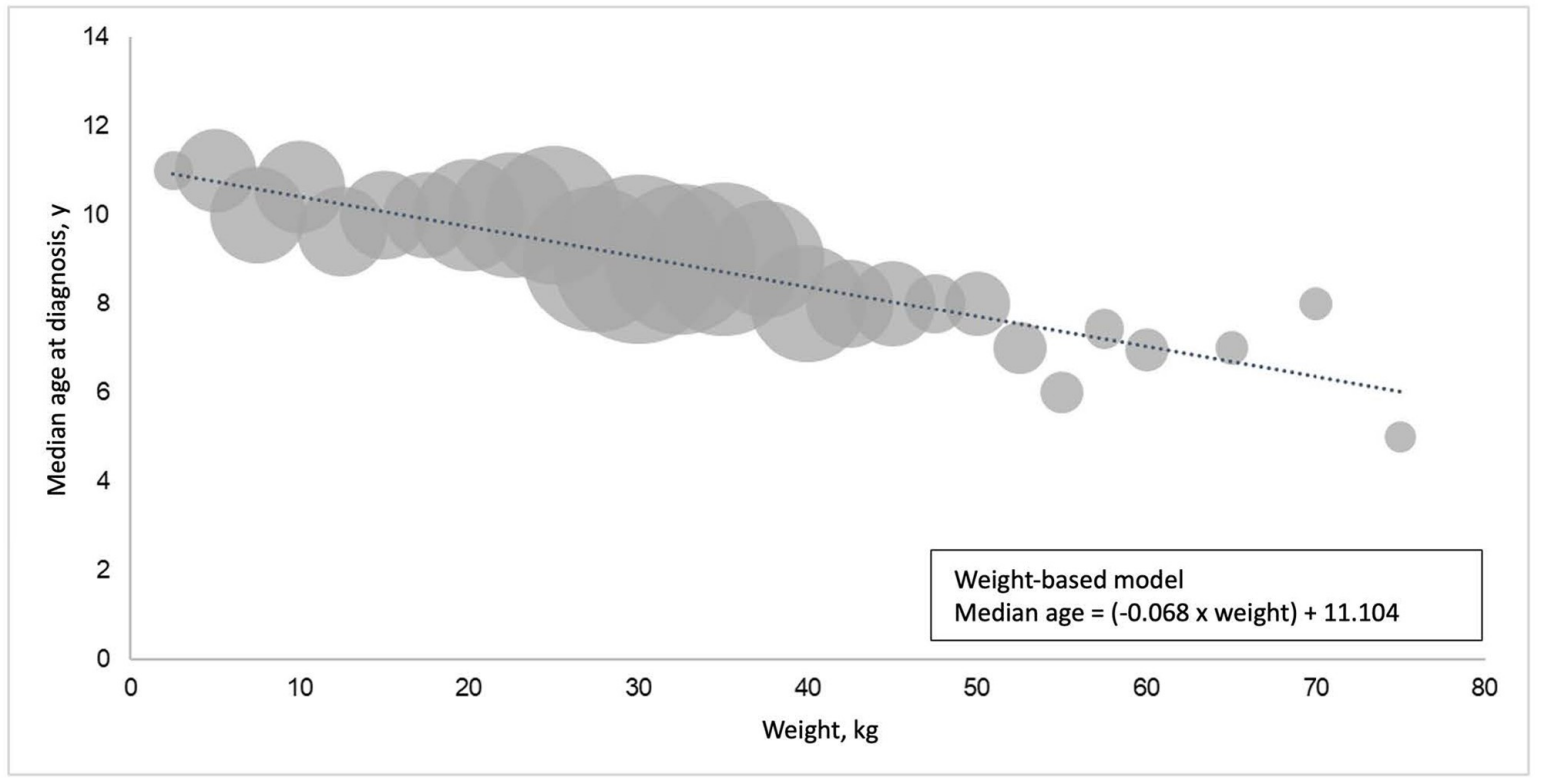
Median age at cancer diagnosis by weight for 2,551 dogs that had a documented weight.

Weights were available for 2,551 dogs. Weights ranged from 2.5–98.0 kg (Fig 4), with a mean of 30.3 kg and a median of 30.6 kg (Table 1). In general, as the weight of the dog increased, the median age at cancer diagnosis decreased; dogs weighing 2.5–5 kg had a median age at cancer diagnosis of 11 years, compared to 5 years for dogs ≥75 kg. By plotting median age at cancer diagnosis for dogs in various weight brackets, a linear regression formula was derived (herein referred to as the weight-based model): median age = (-0.068 × weight) + 11.104 (Fig 5). The coefficient of determination, *R*^2^, for the model was 0.864, indicating high goodness of fit; and the *p*-value for the weight coefficient was <0.0001, indicating high significance for weight in predicting median age at diagnosis.

**Fig 6:**
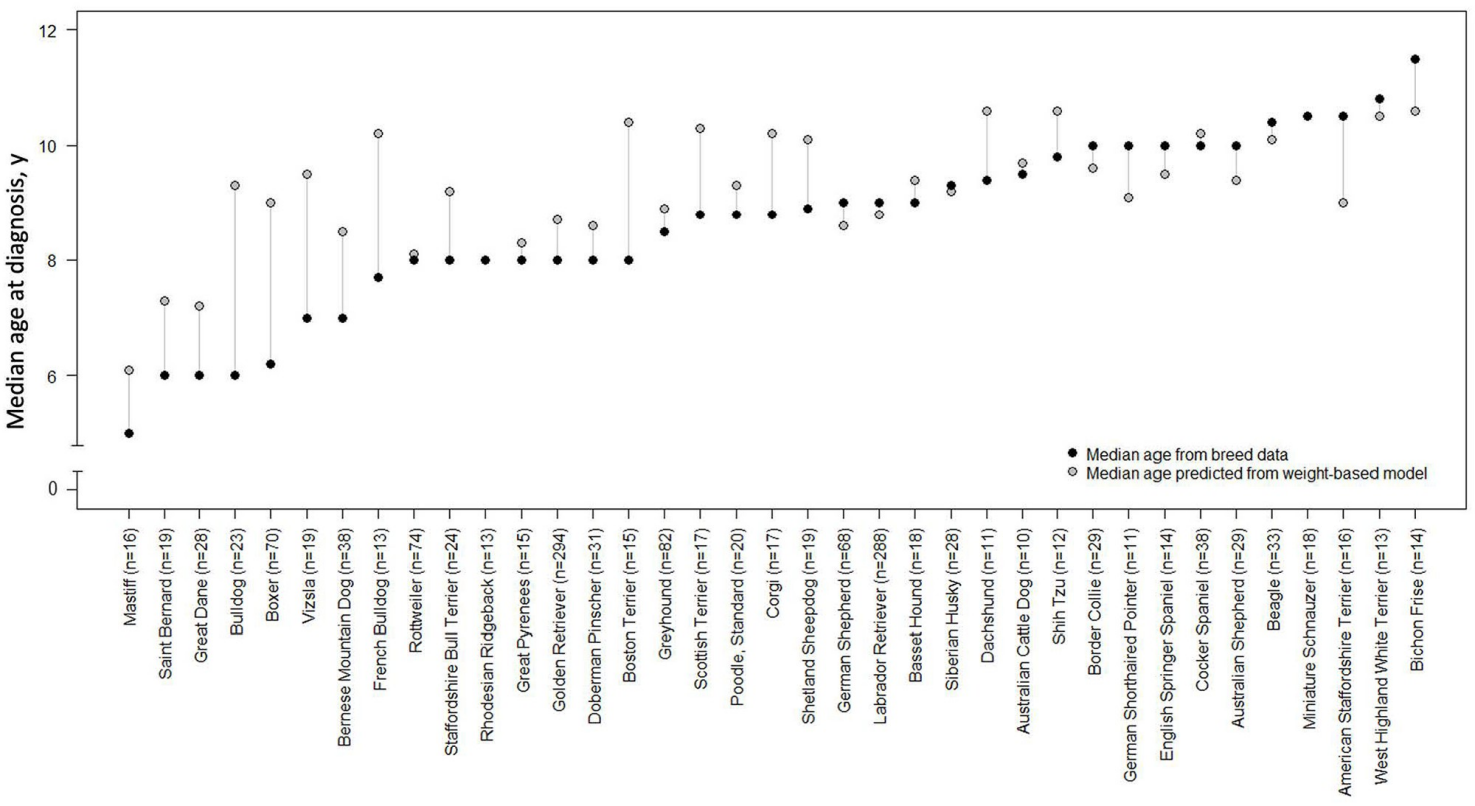
Median age at cancer diagnosis in 1,495 purebred dogs: Breed-based data versus prediction from weight-based model for breeds that had a documented weight for ≥10 dogs.

Weights were available for ≥10 dogs per breed in 37 breeds in the study population. We calculated the median weight of dogs in each breed; then, the median age at cancer diagnosis in that breed was compared to the predicted median age at cancer diagnosis using the weight-based model (Fig 5). For most breeds (23 of 37), the median age at cancer diagnosis predicted by the weight-based model was within one year of the actual median age calculated from dogs representing that breed. For certain breeds, particularly Bulldogs, Boxers, Vizslas, French Bulldogs, and Boston Terriers, the median age at cancer diagnosis calculated directly from dogs of that breed was >2 years younger than the median age predicted by the weight-based model (Fig 6).

### Age at cancer diagnosis by sex and neuter status

In the overall study population (*n* = 3,452), the age of cancer diagnosis in males was significantly younger than in females (mean 8.3 vs. 8.7 years; *p* <0.0001). When the data were subdivided by sex and neuter status, castrated males were diagnosed with cancer at younger ages than spayed females (mean 8.5 vs. 8.9 years; *p* = 0.0002); however, there was no significant difference between the age at cancer diagnosis for intact males vs. intact females (mean 7.6 vs. 7.3 years; *p* = 0.262). There was a significant difference between castrated vs. intact males (mean 8.5 vs. 7.6 years; *p* <0.0001) and spayed vs. intact females (mean 8.9 vs. 7.3 years; *p* <0.0001), with neutered dogs showing a significantly later mean age at diagnosis than their intact counterparts (Table 3). No interaction was observed between weight and sex/neuter status (*p* = 0.0851).

**Table 3:** Age at cancer diagnosis by sex and neuter status of the study population.

Comparison groups, *n*	Median age at cancer diagnosis, y	Mean age at cancer diagnosis, y	*p*
M: F (1,900: 1,552)	8.4: 9.0	8.3: 8.7	*p* <0.0001
CM: SF (1,452: 1,390)	8.9: 9.0	8.5: 8.9	*p* = 0.0002
IM: IF (446: 161)	7.9: 7.3	7.6: 7.3	*p* = 0.2800
CM: IM (1,452: 446)	8.9: 7.9	8.5: 7.6	*p* <0.0001
SF: IF (1,390: 161)	9.0: 7.3	8.9: 7.3	*p* <0.0001

CM = castrated male; F = female; I = intact; M = male; SF = spayed female; Neuter status was unavailable for 2 males and 1 female.

### Age at cancer diagnosis for common cancer types

**Fig 7:**
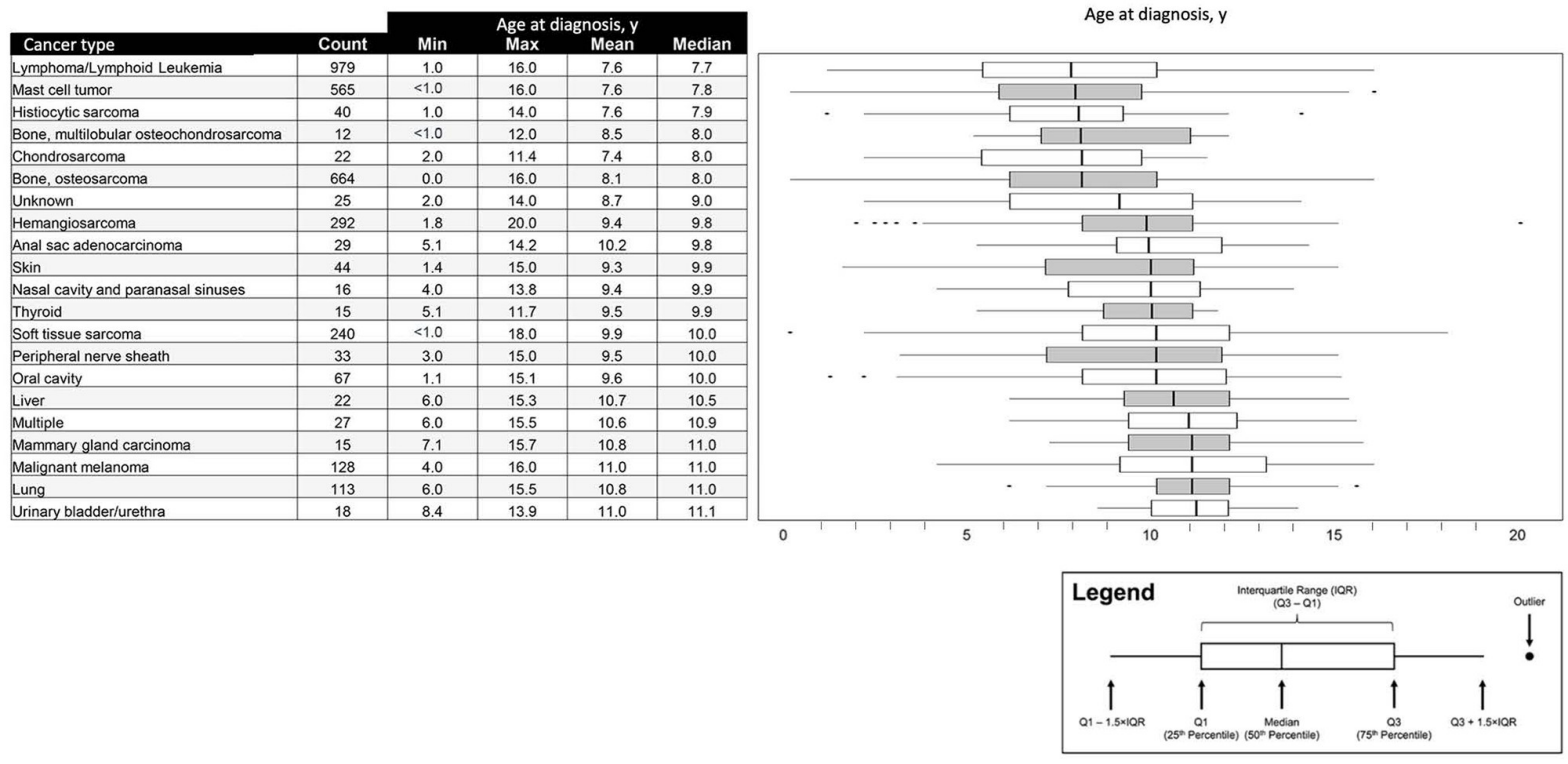
Age distribution at cancer diagnosis for cancer types represented by ≥10 dogs.

The median age at cancer diagnosis was analyzed for cancer types represented by ≥10 dogs (*n* = 21 cancer types). Lymphoma or lymphoid leukemia, mast cell tumor, and histiocytic sarcoma all had median ages at diagnosis <8 years; whereas malignant melanoma and cancers of the mammary gland, lung, and urinary bladder or urethra had median ages at diagnosis of ≥11 years (Fig 7).

## Discussion

The ages at cancer diagnosis in a population of over 3,400 dogs ranged from <1–20 years, with a median of 8.8 years. Overall, in this study population, males were diagnosed with cancer at younger ages than females, and dogs that had been neutered were diagnosed at significantly later ages than their intact counterparts.

When age at cancer diagnosis was analyzed by cancer type, the mean and median ages at cancer diagnosis were found to vary significantly across cancer types, with hematologic malignancies and mast cell tumors being diagnosed at much younger ages than malignant melanomas and lung cancers. These findings are consistent with the literature, wherein the median age at cancer diagnosis for lymphoma has been reported as 6–9 years [32]; oral malignant melanoma [33] and pulmonary tumors [34] are primarily diseases of older dogs, with a reported median age at diagnosis of 11 years. Furthermore, the mean ages at diagnosis for 4 common cancers (hemangiosarcoma, lymphoma, mast cell tumor, osteosarcoma) in our study are closely aligned with findings from a large study at an academic veterinary center in California, USA [35].

The lifetime prevalence of common cancers has been reported to be similar for purebred and mixed-breed dogs, when matched for age, sex, and weight [35]. We found that cancer was diagnosed in purebred dogs at significantly younger ages than mixed-breed dogs. Our finding could be explained in part by selective breeding methods, which may perpetuate germline mutations that predispose certain breeds to cancer at younger ages [3]. However, it should be noted that there was wide variability in the age at diagnosis by breed in the purebred cohort in our study, with median ages at diagnosis of 6.0–11.5 years (in breeds represented by ≥10 dogs).

Weight appeared to be inversely related to age at cancer diagnosis in our overall study population; many of the breeds with younger ages at cancer diagnosis were large- and giant-breed dogs. These findings align with observations from an analysis of cancer claims in >1.6 million dogs covered by a leading US pet health insurer over a 6-year period [36]. If a dog’s weight is known, our weight-based model can be used to estimate the median age at cancer diagnosis; this may be particularly helpful for mixed-breed dogs, or for purebred dogs that have an insufficient number of dogs in this study (<10) to calculate a breed-based median age at diagnosis.

When breed-based calculations were compared to the weight-based model, the median age at cancer diagnosis was similar (within one year) for most breeds, but certain breeds had significant deviations; in particular, Bulldogs, Boxers, Vizslas, French Bulldogs, and Boston Terriers had median ages at cancer diagnosis ≥2 years younger than the weight-predicted ages. This suggests that genetics may play a stronger role in cancer onset in certain breeds, resulting in younger ages at diagnosis. Our finding also aligns with a large study of cancer claims for purebred dogs [4], which found significant differences among breeds for both overall relative cancer risk and for average age at first cancer claim. Extensive similarities were noted between the findings of that analysis and those of our study; for example, Boxers, Great Danes, and French Bulldogs had significantly younger median ages at cancer diagnosis (and average ages at first cancer claim) compared to Beagles, Miniature Schnauzers, and Shih Tzus.[4]

Once an approximate age at which cancer may be diagnosed in each dog is calculated, based on breed-specific data or the weight-based model, an age to initiate screening can be considered.

Cancer screening is based on the premise that cancer develops over time. Cancer progression timelines are well established in human oncology for various types of cancer and are used to inform recommendations for appropriate screening intervals [5, 37–39]. An analysis estimated a latency of 2.2–35.7 years for lymphoproliferative and hematopoietic cancers, and 6.6 to 57 years for solid malignancies, with 35 of the 44 cancer types in the analysis found to progress silently for ≥10 years before detection [40]. Other studies, focusing on genomic evolution timelines across many human cancers, have similarly shown that driver mutations often precede diagnosis by many years to decades [41]. Analyses focusing on specific cancer types have demonstrated that it takes ~17 years for a large benign tumor to evolve into advanced colorectal cancer, but less than 2 additional years for cells within that advanced cancer to acquire metastatic potential [42]; and in pancreatic cancer, ~20 years will pass from the initiation of tumorigenesis until end-stage disease, with metastasis occurring only within the last 2–3 years [43]. In the recurrence setting, a long-term follow-up study in breast cancer showed that recurrence occurred in ~25% of patients at distant sites up to 20 years after the initial curative-intent treatment [44].

These observations regarding the clinical timeline of cancer development are consistent with tumor growth estimates based on reported tumor doubling times, which have been studied extensively in human cancer. Doubling times of 30–300 or more days have been reported for many common cancer types, with significant variation noted across tumor stages, tumor types, and individual patients [45–51]. It is generally accepted that a malignant mass becomes clinically detectable (on physical exam or imaging) once it reaches a volume of ~1 cm^3^ (1.2-cm diameter), at which point it contains upwards of 1 billion cells and weighs approximately 1 gram [52–55]. Using these doubling times, corresponding latency times of 4–25 years can be calculated for various human cancers, which are consistent with the observed clinical timeline of cancer development in humans.

Biologically, progression of cancer over an extended period of time is also likely to occur in dogs, albeit on a shorter timescale than in humans given the compressed canine lifespan [38]. The presence of preclinical malignancy in significant numbers of canine patients has been extensively documented in studies of incidental findings on imaging, surgery, and postmortem examination [56–63].

Studies of spontaneous and induced canine cancer models have provided estimates of *in vivo* tumor doubling times ranging widely from several days to >100 days, depending on tumor type and method of measurement, and varying widely across individuals [64–68]. These doubling times would correspond to latency periods of 1–3 years. However, these estimates are likely conservative given that cancer is not typically diagnosed as soon as it reaches the threshold of clinical detection; in dogs, cancers are often diagnosed, or present for treatment, at 2.5–10 cm [61, 69–78], corresponding to 10 billion to 500 billion cells, and implying latency periods upwards of 5 years. This estimate is consistent with multi-year latency periods documented in dogs following exposure to ionizing radiation: 2–10+ years for bone malignancies [79–81], 2–4 years for hemangiosarcomas [67], 4–10+ years for hepatic malignancies [82], and 3–10+ years for pulmonary malignancies [83].

It is also important to note that tumor growth is not linear during the course of cancer progression. Growth tends to be rapid very early in the disease process but slows considerably by the time the disease reaches a clinically detectable size. This progressive increase in the tumor doubling time as the tumor gets bigger is described by Gompertzian growth kinetics [84, 85] and is recognized as a feature of both human [86–90] and canine [91, 92] malignancies. This non-linear growth trajectory further supports the value of general screening, as it implies a relatively long period when the presence of preclinical but detectable cancer could be confirmed by standard clinical evaluation methods, following a positive screening result.

The relatively long duration of cancer progression, in humans and in dogs, affords multiple opportunities for earlier detection over the lifespan through screening at regular intervals [48, 49, 53, 55, 93, 94]. In humans, it is recommended to start screening for cancer prior to the age of peak incidence of cancer diagnoses, as noted in breast cancer, where peak incidence occurs in the age group 55–64 [95], and annual or biennial screening mammograms are recommended starting at age 45–50 (or earlier ages for high-risk individuals) [6, 96]; or in prostate cancer, where peak incidence occurs in the age group 65–74 [95] and annual or biennial screening is advised to start at age 50 (or earlier ages for high-risk individuals) [6]. Large-scale longitudinal studies are needed to definitively establish the optimal timing and interval of cancer screening in dogs. One such study, the Cancer Lifetime Assessment Screening Study in Canines (CLASSiC) was launched in December 2021 (PetDx, La Jolla, CA); the study aims to prospectively follow over 1,000 initially cancer-free dogs, with semi-annual liquid biopsy testing and comprehensive documentation of cancer-related clinical outcomes, over many years [97, 98].

Based on the knowledge that cancer develops over time and the preclinical phase of cancer in dogs may span years, it might be reasonable to initiate annual cancer screening in dogs starting 2 years prior to the median age at cancer diagnosis for dogs of similar breed or weight. In our study, the median age at diagnosis was ~9 years (8.8 years), supporting a recommended screening age of 7 for all dogs. For dogs belonging to breeds with an earlier median age at cancer diagnosis (6–7 years), screening should begin as early as age 4. We found that 2,012 of 3,452 (58.3%) dogs were diagnosed with cancer at or before age 9. Indeed, even in breeds with a median age at diagnosis of ≥10, 108 of 284 (38.0%) dogs were diagnosed at or before age 9, reinforcing the benefits of starting to screen no later than age 7, even in those breeds.

Our recommendation would align with a screening program centered around a dog’s annual or semiannual wellness visit [93], with serial testing to increase the opportunity for early detection and intervention. In human cancer screening, the value of repeat (interval) testing is well documented, given that it results in a higher cumulative (lifetime) detection rate compared to a single testing event, as each successive test provides an additional opportunity for detection [99–102]. A similar scenario is likely to be observed in cancer screening programs for canine patients. Of note, each testing instance also provides an additional opportunity for a false positive to be reported, increasing the probability that a given patient will receive a false positive result at some point during a multi-year period of regular screening [103]. This underscores the importance of using a screening test with a high specificity (low false positive rate).

The strengths of our study include the large size of the overall cohort and the wide range of breeds and cancer types represented; however, there are also several limitations. For dogs from cohort 2, the dog’s age at collection was used as a proxy for age at cancer diagnosis. In doing so, the actual age at diagnosis is likely overestimated, to an unknown extent (possibly by weeks or months). It should be noted that cohort 2 dogs had their age at collection reported in years, rather than years and months, potentially offsetting some of this putative overestimation.

Additionally, the dogs from cohorts 2 and 3 represented a skewed distribution of cancer types. The collection of dogs for cohort 2 was focused primarily on enrolling 7 pre-defined cancer types (though enrollment of other cancer types was not prohibited); and cohort 3 only provided data for 4 cancer types. These selection biases may have enriched our study for dogs with certain demographic characteristics, because particular cancer types may disproportionately affect dogs of certain breeds, weights, or ages, and may have also impacted the estimate for median age at cancer diagnosis for a given breed, if certain cancers were under- or over-represented for that breed in the cohorts 2 and 3 datasets.

The three cohorts that contributed data to the current study were enrolled at different timepoints. This represents another limitation of our study, as year of diagnosis may have impacted the type(s) of diagnostic testing available at that time, as well as the willingness to pursue testing.

For the cohort of purebred dogs, the median age at cancer diagnosis was calculated for breeds represented by ≥10 dogs. It is not clear if this is a sufficient number of dogs for deriving a valid median age at cancer diagnosis for each of these breeds. More accurate determinations are expected in the future, as data from larger numbers of purebred dogs are collected to inform each of the breed-based estimates.

Another limitation is that breed assignments were provided by the enrolling site, with no way to ensure the accuracy of this information. Furthermore, approximately 2% of dogs were assigned a breed of “other”; an undefined number of these dogs could have been purebred.

We estimate that >95% of dogs in our study were from the United States. This may limit the generalizability of the study findings to other regions of the world in which different environmental characteristics, neuter practices, breed distributions, or other considerations may play a role in age at cancer diagnosis.

## Conclusions

Liquid biopsy opens new opportunities for earlier cancer detection in veterinary medicine through convenient screening at regular intervals. The current study provides important reference data to help inform the optimal age at which initiation of cancer screening might be considered in dogs of various breeds and weights. Such information may guide the incorporation of blood-based cancer screening into routine wellness evaluations for individual dogs, with the potential to improve the ability of veterinarians to detect cancer in the preclinical phase when the disease is more manageable.

## Supporting information

S1 Table Demographics/Characteristics of the study population of 3,452 client-owned cancer-diagnosed dogs, and the percent contributions of the three cohorts that provided data for the current study. (DOCX)

S2 Table Cancer types represented in the study population of 3,452 dogs, and the percent contributions of the three cohorts that provided data for the current study. (DOCX)

S3 Table Full subject level data for dogs in the study population (n = 3,452). (DOCX)

S4 Table Age at cancer diagnosis (range, mean, and median) by breed. (DOCX)
