# Supplementary material for: Age at cancer diagnosis by breed, weight, sex, and cancer type in a cohort of more than 3,000 dogs: Determining the optimal age to initiate cancer screening in canine patients
Source: PLoS One. 2023 Feb 1;18(2):e0280795. doi: 10.1371/journal.pone.0280795 (PMC9891508; doi:10.1371/journal.pone.0280795)
Supplement: S1 Table — (DOCX) [file pone.0280795.s001.docx]

**S1 Table. Demographics/characteristics of the study population of 3,452 client-owned cancer-diagnosed dogs, and the percent contributions of the three cohorts that provided data for the current study.**

|  | | Overall study population | Cohort 1 | | Cohort 2 | | Cohort 3 | |
| --- | --- | --- | --- | --- | --- | --- | --- | --- |
| Characteristics | | n=3,452 | n=663 | Percentage of overall study population contributed by Cohort 1 | n=1,888 | Percentage of overall study population contributed by Cohort 2 | n=901 | Percentage of overall study population contributed by Cohort 3 |
| Breed | Purebred | 2,537 | 345 | 13.6% | 1,415 | 55.8% | 777 | 30.6% |
|  | Mixed-breed or other | 915 | 318 | 34.8% | 473 | 51.7% | 124 | 13.5% |
| Sex | Male | 1,900 | 355 | 18.7% | 996 | 52.4% | 549 | 28.9% |
|  | Castrated | 1,452 | 310 | 21.3% | 877 | 60.4% | 265 | 18.3% |
|  | Intact | 446 | 43 | 9.6% | 119 | 26.7% | 284 | 63.7% |
|  | Status not provided | 2 | 2 | 100.0% | 0 | 0.0% | 0 | 0.0% |
|  | Female | 1,552 | 308 | 19.8% | 892 | 57.5% | 352 | 22.7% |
|  | Spayed | 1,390 | 283 | 20.3% | 853 | 61.4% | 254 | 18.3% |
|  | Intact | 161 | 24 | 14.9% | 39 | 24.2% | 98 | 60.9% |
|  | Status not provided | 1 | 1 | 100.0% | 0 | 0.0% | 0 | 0.0% |
| Weight | Range | 2.5-98.0 kg | 4.5-81.5 kg |  | 2.5-98.0 kg |  | N/A |  |
|  | Mean | 30.3 kg | 26.6 kg |  | 31.6 kg |  | N/A |  |
|  | Median | 30.6 kg | 27.2 kg |  | 31.8 kg |  | N/A |  |
| Age | Range | <1.0-20.0 years | <1.0-15.9 years |  | <1.0-20.0 years |  | 1.0-11.9 years |  |
|  | Mean | 8.5 years | 9.3 years |  | 8.8 years |  | 7.2 years |  |
|  | Median | 8.8 years | 9.5 years |  | 9.0 years |  | 7.3 years |  |
