# Supplementary material for: Age at cancer diagnosis by breed, weight, sex, and cancer type in a cohort of more than 3,000 dogs: Determining the optimal age to initiate cancer screening in canine patients
Source: PLoS One. 2023 Feb 1;18(2):e0280795. doi: 10.1371/journal.pone.0280795 (PMC9891508; doi:10.1371/journal.pone.0280795)
Supplement: S2 Table — (DOCX) [file pone.0280795.s002.docx]

**S2 Table. Cancer types represented in the study population of 3,452 dogs, and the percent contributions of the three cohorts that provided data for the current study.**

| Cancer type and/or location | Overall study population  (Number of dogs) | Cohort 1  (Number of dogs) | Percentage of each cancer type contributed by Cohort 1 | Cohort 2  (Number of dogs) | Percentage of each cancer type contributed by Cohort 2 | Cohort 3  (Number of dogs) | Percentage of each cancer type contributed by Cohort 3 |
| --- | --- | --- | --- | --- | --- | --- | --- |
| Lymphoma/lymphoid leukemia | 979 | 159 | 16.2% | 454 | 46.4% | 366 | 37.4% |
| Bone, osteosarcoma | 664 | 56 | 8.4% | 484 | 72.9% | 124 | 18.7% |
| Mast cell tumor | 565 | 82 | 14.5% | 187 | 33.1% | 296 | 52.4% |
| Hemangiosarcoma | 292 | 17 | 5.8% | 163 | 55.8% | 112 | 38.4% |
| Soft tissue sarcoma | 240 | 57 | 23.8% | 183 | 76.3% | 0 | 0.0% |
| Malignant melanoma | 128 | 19 | 14.8% | 109 | 85.2% | 0 | 0.0% |
| Lung | 113 | 12 | 10.6% | 101 | 89.4% | 0 | 0.0% |
| Oral cavity | 67 | 34 | 50.7% | 33 | 49.3% | 0 | 0.0% |
| Skin | 44 | 21 | 47.7% | 23 | 52.3% | 0 | 0.0% |
| Histiocytic sarcoma | 40 | 11 | 27.5% | 29 | 72.5% | 0 | 0.0% |
| Peripheral nerve sheath | 33 | 3 | 9.1% | 30 | 90.9% | 0 | 0.0% |
| Anal sac adenocarcinoma | 29 | 28 | 96.6% | 1 | 3.4% | 0 | 0.0% |
| Multiple concurrent primary cancers | 27 | 24 | 88.9% | 0 | 0.0% | 3 | 11.1% |
| Unknown* | 25 | 0 | 0.0% | 25 | 100.0% | 0 | 0.0% |
| Chondrosarcoma | 22 | 7 | 31.8% | 15 | 68.2% | 0 | 0.0% |
| Liver | 22 | 14 | 63.6% | 8 | 36.4% | 0 | 0.0% |
| Urinary bladder/urethra | 18 | 17 | 94.4% | 1 | 5.6% | 0 | 0.0% |
| Nasal cavity and paranasal sinuses | 16 | 14 | 87.5% | 2 | 12.5% | 0 | 0.0% |
| Mammary gland carcinoma | 15 | 13 | 86.7% | 2 | 13.3% | 0 | 0.0% |
| Thyroid | 15 | 14 | 93.3% | 1 | 6.7% | 0 | 0.0% |
| Bone, multilobular osteochondrosarcoma | 12 | 4 | 33.3% | 8 | 66.7% | 0 | 0.0% |
| Bone, fibrosarcoma | 9 | 2 | 22.2% | 7 | 77.8% | 0 | 0.0% |
| Adrenal gland | 8 | 6 | 75.0% | 2 | 25.0% | 0 | 0.0% |
| Bone, sarcoma (other) | 8 | 3 | 37.5% | 5 | 62.5% | 0 | 0.0% |
| Brain | 8 | 8 | 100.0% | 0 | 0.0% | 0 | 0.0% |
| Spleen | 8 | 3 | 37.5% | 5 | 62.5% | 0 | 0.0% |
| Kidney | 6 | 3 | 50.0% | 3 | 50.0% | 0 | 0.0% |
| Small intestine | 6 | 3 | 50.0% | 3 | 50.0% | 0 | 0.0% |
| Prostate | 5 | 5 | 100.0% | 0 | 0.0% | 0 | 0.0% |
| Transmissible venereal tumor | 5 | 5 | 100.0% | 0 | 0.0% | 0 | 0.0% |
| Heart base | 3 | 3 | 100.0% | 0 | 0.0% | 0 | 0.0% |
| Pancreas | 3 | 3 | 100.0% | 0 | 0.0% | 0 | 0.0% |
| Bile duct | 2 | 0 | 0.0% | 2 | 100.0% | 0 | 0.0% |
| Mediastinum | 2 | 1 | 50.0% | 1 | 50.0% | 0 | 0.0% |
| Multiple myeloma | 2 | 2 | 100.0% | 0 | 0.0% | 0 | 0.0% |
| Salivary gland | 2 | 2 | 100.0% | 0 | 0.0% | 0 | 0.0% |
| Spinal cord | 2 | 2 | 100.0% | 0 | 0.0% | 0 | 0.0% |
| Ear canal | 1 | 1 | 100.0% | 0 | 0.0% | 0 | 0.0% |
| Esophagus | 1 | 1 | 100.0% | 0 | 0.0% | 0 | 0.0% |
| Large intestine | 1 | 1 | 100.0% | 0 | 0.0% | 0 | 0.0% |
| Nasal planum | 1 | 1 | 100.0% | 0 | 0.0% | 0 | 0.0% |
| Thymoma | 1 | 1 | 100.0% | 0 | 0.0% | 0 | 0.0% |
| Uterus | 1 | 0 | 0.0% | 1 | 100.0% | 0 | 0.0% |
| Vagina | 1 | 1 | 100.0% | 0 | 0.0% | 0 | 0.0% |
