# Supplementary material for: Age at cancer diagnosis by breed, weight, sex, and cancer type in a cohort of more than 3,000 dogs: Determining the optimal age to initiate cancer screening in canine patients
Source: PLoS One. 2023 Feb 1;18(2):e0280795. doi: 10.1371/journal.pone.0280795 (PMC9891508; doi:10.1371/journal.pone.0280795)
Supplement: S4 Table — (DOCX) [file pone.0280795.s004.docx]

**S4 Table: Age at cancer diagnosis (range, mean, and median) by breed.**

| Breed | Number of subjects in study population | Youngest age at diagnosis in study population  (years) | Oldest age at diagnosis in study population (years) | Mean age at diagnosis in study population  (years) | Median age at diagnosis in study population  (years) |
| --- | --- | --- | --- | --- | --- |
| Affenpinscher | 1 | 10.0 | 10.0 | 10.0 | 10.0 |
| Afghan Hound | 1 | 2.0 | 2.0 | 2.0 | 2.0 |
| Airedale Terrier | 7 | 1.0 | 11.0 | 7.9 | 8.0 |
| Akbash | 1 | 2.0 | 2.0 | 2.0 | 2.0 |
| Akita | 6 | 2.0 | 10.0 | 6.3 | 6.5 |
| Alaskan Malamute | 4 | 7.0 | 13.0 | 10.5 | 11.0 |
| American Eskimo Dog | 6 | 9.5 | 15.3 | 11.8 | 11.0 |
| American Foxhound | 1 | 10.0 | 10.0 | 10.0 | 10.0 |
| American Staffordshire Terrier | 16 | 1.0 | 13.0 | 9.1 | 10.5 |
| American Water Spaniel | 2 | 5.0 | 5.0 | 5.0 | 5.0 |
| Anatolian Shepherd | 5 | 3.7 | 11.0 | 6.5 | 5.0 |
| Australian Cattle Dog | 16 | 6.0 | 14.5 | 9.3 | 9.5 |
| Australian Shepherd | 52 | 1.3 | 16.0 | 9.4 | 10.0 |
| Barbet | 1 | 7.0 | 7.0 | 7.0 | 7.0 |
| Basenji | 2 | 10.0 | 10.3 | 10.2 | 10.2 |
| Basset Hound | 18 | 6.0 | 13.0 | 9.2 | 9.0 |
| Beagle | 38 | 3.6 | 15.0 | 10.1 | 10.4 |
| Bearded Collie | 1 | 9.7 | 9.7 | 9.7 | 9.7 |
| Belgian Malinois | 3 | 0.9 | 9.0 | 6.3 | 9.0 |
| Belgian Sheepdog | 3 | 6.0 | 13.0 | 10.3 | 12.0 |
| Belgian Tervuren | 1 | 6.0 | 6.0 | 6.0 | 6.0 |
| Bernese Mountain Dog | 59 | 2.3 | 12.0 | 6.9 | 7.0 |
| Bichon Frise | 14 | 5.0 | 15.1 | 11.3 | 11.5 |
| Black and Tan Coonhound | 4 | 4.0 | 10.0 | 7.8 | 8.5 |
| Bloodhound | 4 | 4.0 | 12.0 | 7.5 | 7.0 |
| Border Collie | 44 | 2.4 | 13.8 | 9.1 | 10.0 |
| Border Terrier | 1 | 8.0 | 8.0 | 8.0 | 8.0 |
| Boston Terrier | 27 | 1.9 | 14.0 | 8.0 | 8.0 |
| Bouvier des Flandres | 3 | 8.0 | 13.0 | 9.7 | 8.0 |
| Boxer | 178 | 1.0 | 12.0 | 6.2 | 6.2 |
| Briard | 5 | 7.0 | 12.0 | 9.4 | 9.0 |
| Brittany | 5 | 8.0 | 11.6 | 9.7 | 9.0 |
| Bull Terrier | 2 | 4.0 | 6.8 | 5.4 | 5.4 |
| Bulldog | 53 | 1.3 | 12.0 | 6.0 | 6.0 |
| Bullmastiff | 5 | 4.0 | 9.0 | 6.0 | 5.0 |
| Cairn Terrier | 7 | 11.0 | 15.0 | 12.8 | 12.9 |
| Cane Corso | 2 | 5.0 | 7.7 | 6.4 | 6.4 |
| Corgi | 29 | 3.2 | 14.0 | 8.7 | 8.8 |
| Cavalier King Charles Spaniel | 5 | 6.8 | 13.6 | 8.8 | 7.5 |
| Chesapeake Bay Retriever | 6 | 7.0 | 13.0 | 9.7 | 10.0 |
| Chihuahua | 19 | 2.7 | 13.0 | 8.2 | 8.2 |
| Chinese Shar-Pei | 4 | 5.0 | 9.0 | 6.3 | 5.5 |
| Chow Chow | 7 | 7.0 | 20.0 | 11.4 | 9.0 |
| Cocker Spaniel | 51 | 2.0 | 16.0 | 9.0 | 10.0 |
| Collie | 16 | 2.0 | 11.0 | 8.3 | 9.0 |
| Curly-Coated Retriever | 1 | 13.0 | 13.0 | 13.0 | 13.0 |
| Dachshund | 16 | 3.6 | 15.0 | 9.5 | 9.4 |
| Dalmatian | 4 | 7.0 | 12.0 | 10.3 | 11.1 |
| Doberman Pinscher | 43 | 2.0 | 12.0 | 7.6 | 8.0 |
| Dogo Argentino | 1 | 8.8 | 8.8 | 8.8 | 8.8 |
| Dogue de Bordeaux | 1 | 7.0 | 7.0 | 7.0 | 7.0 |
| English Cocker Spaniel | 6 | 5.0 | 14.0 | 9.3 | 9.0 |
| English Setter | 4 | 10.0 | 13.0 | 11.0 | 10.5 |
| English Springer Spaniel | 22 | 6.0 | 14.0 | 10.0 | 10.0 |
| Flat-Coated Retriever | 5 | 7.7 | 13.0 | 9.7 | 8.9 |
| French Bulldog | 13 | 2.9 | 12.8 | 7.5 | 7.7 |
| German Shepherd | 102 | 1.2 | 14.0 | 8.4 | 9.0 |
| German Shorthaired Pointer | 11 | 3.0 | 12.0 | 8.8 | 10.0 |
| Giant Schnauzer | 6 | 5.0 | 12.0 | 7.8 | 8.0 |
| Golden Retriever | 422 | 1.0 | 16.0 | 8.2 | 8.0 |
| Gordon Setter | 3 | 10.0 | 10.0 | 10.0 | 10.0 |
| Great Dane | 42 | <1.0 | 11.0 | 6.0 | 6.0 |
| Great Pyrenees | 15 | 2.0 | 12.0 | 7.6 | 8.0 |
| Greater Swiss Mountain Dog | 1 | 7.2 | 7.2 | 7.2 | 7.2 |
| Greyhound | 82 | <1.0 | 14.0 | 8.2 | 8.5 |
| Havanese | 1 | 13.6 | 13.6 | 13.6 | 13.6 |
| Irish Setter | 4 | 5.0 | 12.0 | 9.3 | 10.0 |
| Irish Wolfhound | 18 | 3.2 | 11.0 | 6.3 | 6.1 |
| Italian Greyhound | 2 | 12.0 | 12.0 | 12.0 | 12.0 |
| Jack Russell Terrier | 13 | 1.6 | 11.5 | 7.7 | 8.1 |
| Keeshond | 2 | 9.0 | 13.0 | 11.0 | 11.0 |
| Kerry Blue Terrier | 1 | 7.4 | 7.4 | 7.4 | 7.4 |
| Labrador Retriever | 397 | 1.0 | 15.1 | 8.8 | 9.0 |
| Leonberger | 3 | 3.0 | 7.0 | 4.7 | 4.0 |
| Lhasa Apso | 4 | 8.0 | 12.3 | 10.3 | 10.5 |
| Maltese | 7 | 6.5 | 16.0 | 11.3 | 11.0 |
| Mastiff | 16 | 3.0 | 12.0 | 6.2 | 5.0 |
| Miniature Pinscher | 4 | 4.0 | 13.0 | 8.5 | 8.5 |
| Miniature Schnauzer | 22 | 3.0 | 14.0 | 9.7 | 10.5 |
| Newfoundland | 8 | 3.0 | 10.0 | 6.6 | 6.5 |
| Norwegian Elkhound | 2 | 15.0 | 15.0 | 15.0 | 15.0 |
| Nova Scotia Duck Tolling Retriever | 1 | 7.0 | 7.0 | 7.0 | 7.0 |
| Old English Sheepdog | 8 | 6.0 | 12.0 | 8.3 | 8.0 |
| Otterhound | 2 | 4.0 | 11.0 | 7.5 | 7.5 |
| Papillon | 2 | 1.0 | 11.0 | 6.0 | 6.0 |
| Parson Russell Terrier | 5 | 8.0 | 14.4 | 11.1 | 10.0 |
| Pointer | 1 | 10.0 | 10.0 | 10.0 | 10.0 |
| Pomeranian | 4 | 5.0 | 10.0 | 8.3 | 9.1 |
| Poodle, Miniature | 5 | 5.0 | 12.0 | 9.7 | 10.4 |
| Poodle, Standard | 33 | 2.5 | 13.8 | 8.5 | 8.8 |
| Poodle, Toy | 3 | 1.1 | 14.1 | 8.4 | 10.1 |
| Portuguese Sheepdog | 1 | 9.6 | 9.6 | 9.6 | 9.6 |
| Portuguese Water Dog | 4 | 2.0 | 10.0 | 7.5 | 9.0 |
| Pug | 33 | 2.0 | 11.5 | 7.8 | 8.6 |
| Rat Terrier | 1 | 9.9 | 9.9 | 9.9 | 9.9 |
| Rhodesian Ridgeback | 13 | 4.9 | 11.0 | 8.2 | 8.0 |
| Rottweiler | 168 | 1.6 | 16.0 | 7.6 | 8.0 |
| Russell Terrier | 3 | 11.5 | 13.4 | 12.2 | 11.8 |
| Saint Bernard | 25 | 4.0 | 9.0 | 5.9 | 6.0 |
| Salukis | 1 | 8.7 | 8.7 | 8.7 | 8.7 |
| Samoyed | 4 | 7.0 | 14.0 | 11.0 | 11.5 |
| Schipperke | 1 | 12.0 | 12.0 | 12.0 | 12.0 |
| Scottish Terrier | 17 | 3.0 | 11.0 | 8.3 | 8.8 |
| Shetland Sheepdog | 26 | 2.0 | 15.0 | 8.9 | 8.9 |
| Shih Tzu | 18 | 5.5 | 14.3 | 9.4 | 9.8 |
| Siberian Husky | 28 | 5.0 | 15.7 | 9.8 | 9.3 |
| Small Munsterlander | 1 | 6.0 | 6.0 | 6.0 | 6.0 |
| Smooth Fox Terrier | 1 | 14.0 | 14.0 | 14.0 | 14.0 |
| Soft Coated Wheaten Terrier | 4 | 9.0 | 16.0 | 11.8 | 11.0 |
| Spinone Italiano | 1 | 7.0 | 7.0 | 7.0 | 7.0 |
| Staffordshire Bull Terrier | 24 | 3.0 | 11.0 | 7.9 | 8.0 |
| Standard Schnauzer | 2 | 7.0 | 9.0 | 8.0 | 8.0 |
| Sussex Spaniel | 1 | 7.0 | 7.0 | 7.0 | 7.0 |
| Tibetan Terrier | 1 | 8.0 | 8.0 | 8.0 | 8.0 |
| Toy Fox Terrier | 1 | 9.0 | 9.0 | 9.0 | 9.0 |
| Vizsla | 19 | 3.0 | 12.0 | 7.3 | 7.0 |
| Weimaraner | 9 | 3.0 | 13.0 | 10.3 | 11.0 |
| Welsh Terrier | 1 | 11.0 | 11.0 | 11.0 | 11.0 |
| West Highland White Terrier | 14 | 6.0 | 13.5 | 10.8 | 10.8 |
| Wire Fox Terrier | 1 | 11.0 | 11.0 | 11.0 | 11.0 |
| Yorkshire Terrier | 11 | 3.8 | 11.5 | 8.0 | 8.7 |
| Mixed-Breed | 858 | <1.0 | 18.0 | 9.2 | 9.6 |
| Other | 57 | 3.0 | 16.0 | 9.0 | 9.0 |
